# An oncogenic mutant of RHEB, RHEB Y35N, exhibits an altered interaction with BRAF resulting in cancer transformation

**DOI:** 10.1186/s12885-017-3938-5

**Published:** 2018-01-10

**Authors:** Jeffrey J. Heard, Ivy Phung, Mark I. Potes, Fuyuhiko Tamanoi

**Affiliations:** 1 Department of Microbiology, Immunology, and Molecular Genetics, University of California, 1602 Molecular Sciences Bldg, 609 Charles E. Young Dr. East, Los Angeles, CA 90095-1489 USA; 2 Institute for Integrated Cell-Material Sciences, Kyoto University, Kyoto, Japan

**Keywords:** RHEB, RAF, MEK, ERK, RAF/MEK/ERK, Cancer, Signaling, Transformation, RHEB Y35N

## Abstract

**Background:**

RHEB is a unique member of the RAS superfamily of small GTPases expressed in all tissues and conserved from yeast to humans. Early studies on RHEB indicated a possible RHEB-RAF interaction, but this has not been fully explored. Recent work on cancer genome databases has revealed a reoccurring mutation in RHEB at the Tyr35 position, and a recent study points to the oncogenic potential of this mutant that involves activation of RAF/MEK/ERK signaling. These developments prompted us to reassess the significance of RHEB effect on RAF, and to compare mutant and wild type RHEB.

**Methods:**

To study RHEB-RAF interaction, and the effect of the Y35N mutation on this interaction, we used transfection, immunoprecipitation, and Western blotting techniques. We generated cell lines stably expressing RHEB WT, RHEB Y35N, and KRAS G12V, and monitored cellular transforming properties through cell proliferation, anchorage independent growth, cell cycle analysis, and foci formation assays.

**Results:**

We observe a strong interaction between RHEB and BRAF, but not with CRAF. This interaction is dependent on an intact RHEB effector domain and RHEB-GTP loading status. RHEB overexpression decreases RAF activation of the RAF/MEK/ERK pathway and RHEB knockdown results in an increase in RAF/MEK/ERK activation. RHEB Y35N mutation has decreased interaction with BRAF, and RHEB Y35N cells exhibit greater BRAF/CRAF heterodimerization resulting in increased RAF/MEK/ERK signaling. This leads to cancer transformation of RHEB Y35N stably expressing cell lines, similar to KRAS G12 V expressing cell lines.

**Conclusions:**

RHEB interaction with BRAF is crucial for inhibiting RAF/MEK/ERK signaling. The RHEB Y35N mutant sustains RAF/MEK/ERK signaling due to a decreased interaction with BRAF, leading to increased BRAF/CRAF heterodimerization. RHEB Y35N expressing cells undergo cancer transformation due to decreased interaction between RHEB and BRAF resulting in overactive RAF/MEK/ERK signaling. Taken together with the previously established function of RHEB to activate mTORC1 signaling, it appears that RHEB performs a dual function; one is to suppress the RAF/MEK/ERK signaling and the other is to activate mTORC1 signaling.

**Electronic supplementary material:**

The online version of this article (10.1186/s12885-017-3938-5) contains supplementary material, which is available to authorized users.

## Background

Ras homolog enriched in brain (RHEB) is a member of the Ras superfamily of small GTPases that are responsible for the activation of numerous important signaling pathways in the cell [1]. RHEB was discovered as a gene expressed in neuronal cells after synaptic stimulation and in the hippocampus after seizures [2]. Later studies revealed RHEB to be expressed ubiquitously in all tissues [3–6]. RHEB is a 21 kDa protein that is 37% identical to KRAS, and shares important features common to small GTPases including five guanine nucleotide binding domains, 6/9 identical amino acids of the Ras effector domain, and a C-terminal CAAX motif that is post-translationally farnesylated [2, 7]. RHEB, like all small GTPases, act as molecular switches in the cell; they switch “on” and activate downstream signaling when bound GTP, and they switch “off” when bound GDP through GTP hydrolysis [7]. However, structural studies have revealed key differences between RHEB and other members of the Ras superfamily of GTPases [8–10]. A conserved amino acid important for GTP hydrolysis, Gln64 (Gln61 in Ras), is buried in a hydrophobic core blocking access to GTP [8]. These unique structural differences cause RHEB to exist in an active GTP-bound state at higher levels than most small GTPases.

Analysis of cancer genomic databases has revealed a reoccurring point mutation in RHEB at tyrosine 35. This mutation has been identified in three patients with clear cell renal cell carcinoma (ccRCC) and two patients with endometrial cancers [11]. RHEB Y35N was found to be significant in ccRCC due to its relatively high mutation rate relative to background and the location of the mutation in an evolutionarily conserved site [11]. Tryosine 35 is present in the highly-conserved effector domain region of small GTPases, a region that facilitates interaction with downstream proteins and signaling activation. It is possible due to the location of this mutation, that it alters RHEB interaction with proteins and therefore alters downstream RHEB signaling pathways. Interestingly, RHEB Y35N exerts transforming effects on NIH3T3 cells as strong as that observed with KRAS G12 V transforming mutant, and this involves ERK signaling [12].

Early studies on RHEB looked at the ability of RHEB to stimulate Ras effectors mainly due to the strong similarities between RHEB and Ras effector domains. It was demonstrated that purified RHEB could interact with RAF-1 in vitro or in a yeast two-hybrid assay [4, 13]. Later studies indicated that RHEB binds BRAF and inhibits BRAF activation of the RAF/MEK/ERK signaling pathway [14–16]. However, biological significance of the RHEB/RAF interaction was not fully explored. Concurrent studies revealed RHEB to activate mTORC1 signaling and the field of RHEB research shifted significantly to the study of mTOR [17, 18]. mTORC1 is a kinase complex that stimulates protein synthesis and cell proliferation [19]. Aberrant RHEB/mTORC1 signaling has been linked to many overgrowth diseases including Lymphangioleiomymoatosis (LAM), Tuberous Sclerosis (TS), Peutz-Jeghers syndrome (PJS) and benign tumor formation [20–22].

We, as well as others, have continued to explore identification of downstream effectors of RHEB, as many GTPases have been shown to interact with multiple downstream effectors [23]. In fact, the presence of multiple downstream effectors is a common feature of the RAS superfamily GTPases. For example, RAS has been shown to activate PI3K, RalGDS, RIN1, RAF, and PKC [24]. Recent publications have linked RHEB to diverse cellular pathways through interactions with AMPK, phospholipase D1 (PLD1), β-secretase (BACE1), PDE4D, and GAPDH [25–29]. Our group recently discovered a novel RHEB interaction with carbamoyl-phosphate synthetase II, aspartate transcarbamoylase, and dihydrooorotase (CAD), resulting in stimulation of pyrimidine nucleotide biosynthesis in the cell [30]. As described in this paper, BRAF can be added as another downstream effector of RHEB.

Above developments concerning RHEB prompted us to re-evaluate RHEB-RAF interaction. In this paper we report a strong interaction between RHEB and BRAF that results in decreased BRAF-CRAF dimerization and decreased RAF/MEK/ERK signaling. This relationship is dependent on an intact effector domain and the GTP loading status of RHEB. Additionally, the Y35N mutation decreases RHEB-BRAF interaction, resulting in increased BRAF-CRAF dimerization and activation of RAF/MEK/ERK signaling. Cell lines stably expressing RHEB Y35N exhibit cancer transformation properties similar to KRAS G12 V. This evidence suggests that RHEB regulates the RAF/MEK/ERK pathway from aberrant overactivation.

## Methods

### Cell culture and transfection

HEK293T and NIH 3 T3 cells were obtained from ATCC (ATCC Numbers CRL-3216 and CRL-1658, respectively). HEK293T and NIH 3T3 cells were maintained in Dulbecco’s modified Eagle’s medium (DMEM) supplemented with 10% (vol/vol) fetal bovine serum and 1% (vol/vol) penicillin/streptomycin. Cells were cultured at 37 °C in a 5% CO_2_ incubator. Transfection was carried out using Lipofectamine 2000 (Invitrogen) according to the manufacturer’s instructions.

### FLAG Immunoprecipitation

HEK293T cells expressing FLAG tagged RHEB -WT, −T38A, -Y35N, -D60I, and KRAS-G12V were immunoprecipitated using anti-FLAG M2 magnetic beads (Sigma). Briefly, the cells were lysed with lysis buffer (50 mM HEPES pH 7.4, 150 mM NaCl, 0.4% CHAPS, 1X Complete EDTA-free protease inhibitor cocktail (Roche), 1 mM Na_3_VO_4_), 150 mM NaCl, 25 mM MgCl_2_), and the supernatant was cleared of cellular debris using centrifugation (16,000×g for 10 min). Cleared supernatant was mixed with anti-FLAG M2 magnetic beads (Sigma) for affinity purification. The beads were collected, washed four times with lysis buffer. The remaining bound proteins were eluted three times with lysis buffer containing 62 μg/mL of 3X FLAG peptide. Eluted proteins were concentrated using Amicon Ultra 0.5-ml centrifugal filters NMWL 10 K (EMD Millipore, Billerica, MA).

### Western blotting & antibodies

The amount of total protein concentration in cellular lysate was determined by Bio-Rad protein assay according to manufacturer’s instructions. Western blotting was carried out as described previously [31]. Briefly, equal protein extracts from samples were separated by SDS-PAGE and transferred onto nitrocellulose membrane (GE Healthcare). The membrane was blocked in 5% bovine serum albumin, incubated in primary antibodies, and followed by incubation in secondary antibodies conjugated to Horseradish peroxidase (HRP). The membrane was incubated in Pierce ECL Western Blotting Substrate solution (Thermo Scientific) to activate the HRP activity, and protein bands were detected on film.

The following antibodies were purchased from Cell Signaling Technologies: Anti –RHEB, -KRAS, -ACTIN, -totalS6, -phosphoS6, -totalERK, -phosphoERK, -BRAF, and –CRAF. Anti-FLAG was purchased from Sigma.

### Generation of Lentivirus and stably expressing cell lines

Stably expressing cell lines were generated using lentiviral transduction method. The RHEB and KRAS G12V genes were amplified from pcDNA.3 plasmid vectors already containing Flag-RHEB or Flag-KRAS G12V via PCR, and primers containing EcoRI and BamHI restriction enzyme cut sites. Amplified products were ligated into the lentiviral transfer plasmid pCCL-c-MCS, after it was digested with EcoRI and BamHI, using ligase. The RHEB Y35N mutation was generated using Quickchange Lighting Site-Directed Mutagenesis Kit (Agilent).

Lentivirus was produced by transfecting the lentiviral transfer plasmid, the packaging plasmid (pCMV-R8.9) and the envelope plasmid (pMDG-VSVG) into HEK 293T cells using Lipofectamine 2000 (Invitrogen) according to the manufacturer’s instructions. The media was collected 48 h later and filtered through a 0.45 μm filter. Lentiviral media was stored at −80 °C until ready for use.

NIH 3T3 cells were grown until 90% confluency before adding a mixture of 50% normal media, 50% lentiviral media, and 8 μg/mL polybrene. Cells were incubated for 48 h before being passaged and grown in normal media. Expression of transduced proteins were monitored via Western blot using anti-FLAG antibodies.

### Growth curve assay

Cells were grown under normal conditions (DMEM containing 10% FBS) or serum starved (DMEM without FBS) and measured at given timepoints using the Cell Counting Kit-8 (Dojindo Molecular Technologies, Inc.) according to manufacturer’s instructions. Briefly, cells were grown in 96 well plates, 10 μl of CCK-8 dye was added to each well containing 100 μl of cell media, cells were incubated for 1 h, and then readings were obtained in triplicates using a Spectramax Plus 384 spectrophotometer (Molecular Devices) at O.D. 450 nm.

### Cell cycle analysis

NIH 3T3 cell lines were trypsinized, washed, and suspended in PBS. Cells were fixed for 1 h at 4 °C in 70% ethanol. After fixation, cells were washed of ethanol and suspended in 500 μl of PBS. 20 μl of RNAase A (10 mg/mL stock) and 25 μl of propidium iodide (1 mg/mL stock) solutions were added and the cells were incubated at 37 °C for 30 min. Cells were analyzed by flow cytometry at the UCLA Flow Cytometry Core.

### Foci formation

NIH 3T3 cell lines were grown under normal growth conditions for 3 weeks, fresh media was added every 2-3 days. Cells were visualized with crystal violet staining method. Briefly, cells were fixed with ice-cold methanol for 10 min on ice. Methanol was removed and the cells were incubated in 0.5% crystal violet solution (0.5 g crystal violet in 100 ml of 25% methanol solution) for 5 min at room temperature. Cells were rinsed with H_2_O until no more color came off in the rinse. For quantification, only those foci that were > than 2.5 mm in diameter were counted.

### Soft agar Colony formation assay

To generate a semi-solid media growth surface for cells, first a 1% and a 0.6% (mass/vol) agar-media solution was made and autoclaved. Then a 0.5% base-layer-matrix was generated by heating up the 1% agar solution until dissolved, and mixing it with normal growth media in a 50:50 ratio. The solution was layered onto a cell culture plate and left to solidify in the cell incubator for 1 h. The 0.6% agar solution was heated until dissolved, and placed in 37 °C H_2_O bath to bring down to cell temperature. The NIH 3T3 cell lines were trypsinized and suspended in normal media and the 0.6% agar solution in a 50:50 ratio (now a 0.3% agar-media-cell solution). The 0.3% agar-media-cell solution was layered on top of the 0.5% solidified base-layer-matrix. Cells were grown in incubator as normal for 3–4 weeks, with small amount of normal media added 1×/week to prevent the gels from drying out. Cells were incubated with Nitro Blue Tetrazolium dye 1 mg/ml stock (tablets purchased from Sigma) overnight at 37 °C. Colonies were visualized using BioRad Imager and counted by eye.

## Results

### RHEB interacts with BRAF not CRAF and the RHEB-BRAF interaction is dependent on the intact effector domain and GTP binding

**Fig. 1:**
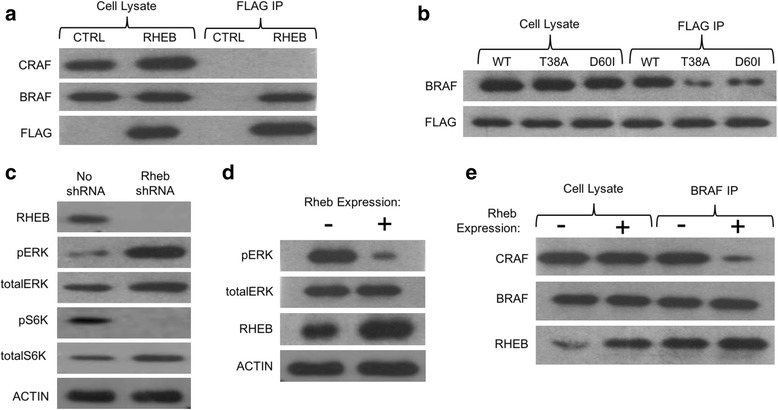
RHEB interacts with BRAF, decreasing BRAF/CRAF heterodimerization and RAF/MEK/ERK signaling. **a** Immunoprecipitation of FLAG-RHEB from HEK 293T cell lysate, and Western blot of CRAF, BRAF, and FLAG. **b** FLAG tagged RHEB WT, T38A, and D60I were expressed in HEK 293T cells. Cell lysates were collected and anti-FLAG immunoprecipitation was performed followed by Western blot for BRAF and FLAG. **c** Western blot showing levels of RHEB, phosphorylated ERK, total ERK, phosphorylated S6K, total S6 K and ACTIN in normal and RHEB knockdown HEK 293T cell lines. Compare the amount of phosphorylated ERK relative to the total amount of ERK. **d** Western blot showing levels of RHEB, phosphorylated ERK, total ERK and ACTIN in normal and overexpressed-RHEB HEK 293T cell lines. Compare the amount of phosphorylated ERK relative to the total amount of ERK. **e** Endogenous BRAF was immunoprecipitated from HEK 293T cells containing endogenous RHEB or overexpressed RHEB. Western blot of RHEB, CRAF, and BRAF is shown

It has been reported that RHEB interacts with RAF kinases, however, reports are conflicting on whether RHEB binds both BRAF and CRAF, or just BRAF [4, 13, 15, 32]. We performed immunoprecipitation of RHEB protein from cells to identify potential interaction between RHEB and RAF. Briefly, plasmids expressing Flag-tagged RHEB were transiently transfected into HEK 293 T cells, and immunoprecipitation using anti-FLAG antibody was performed. We observed a strong interaction with RHEB and BRAF (Fig. 1a). However, we see no interaction between RHEB and CRAF (Fig. 1a).

The RHEB-BRAF interaction is dependent on an intact effector domain. This was examined using the RHEB T38A mutant. The T38A mutation occurs in the RHEB effector domain, and causes decreased interactions between RHEB and its effector proteins such as mTORC1 [18, 33]. As seen in Fig. 1b, we observed BRAF interaction was weaker with the RHEB mutant compared with the wild type, indicating that the RHEB-BRAF interaction is consistent with RHEB effector protein interactions.

Another RHEB mutant we used is RHEB D60I. The mutation occurs in a critical region required for GTP loading, and thus results in a higher amount of inactive RHEB-GDP and decreased RHEB signaling [18, 34]. As shown in Fig. 1b, the RHEB D60I interacted with BRAF less efficiently compared with the wild type, suggesting that the RHEB-BRAF interaction is dependent on RHEB GTP-binding status.

### RHEB inhibits the RAF/MEK/ERK pathway

To test whether this RHEB-BRAF interaction affects the RAF/MEK/ERK pathway, we generated a RHEB knockdown HEK 293T cell line stably expressing RHEB shRNA. ERK activation can be observed through increased levels of phosphorylated Thr202 and Tyr204 of ERK protein. We collected the cell lysate from our RHEB shRNA expressing cell line, and performed a Western blot using an antibody against phosphorylated ERK (Thr202/Tyr204). We observed increased levels of ERK phosphorylation in the RHEB shRNA1 cell lines (Fig. 1c). As a control, we also saw a decrease in the levels of phosphorylated S6 K, indicating inhibition of mTORC1 activity (Fig. 1c).

Additionally, we overexpressed RHEB in HEK 293 T cells and observed a decrease in the levels of phosphorylated ERK (Thr202/Tyr204) in these cells (Fig. 1d). These results strongly suggest that RHEB inhibits the RAF/MEK/ERK pathway.

### RHEB inhibits BRAF/CRAF Heterodimerization

We tested how the interaction between RHEB and BRAF could decrease the RAF/MEK/ERK pathway. It is known that BRAF homodimerization, or heterodimerization with CRAF, is required for activation of the RAF/MEK/ERK pathway [35–37]. It was previously suggested that RHEB-RAF interaction could disrupt BRAF/CRAF heterodimerization [15]. To test this hypothesis we monitored changes in the levels of BRAF-CRAF dimerization in cells with different levels of RHEB expression. We performed immunoprecipitation of BRAF followed by Western blot for CRAF to observe the amount of BRAF complexed with CRAF. BRAF-CRAF dimerization was significantly decreased in the presence of overexpressed RHEB (Fig. 1e). This suggests that RHEB prevents BRAF-CRAF dimerization, thus leading to decreased RAF/MEK/ERK activation.

### RHEB Y35N binds BRAF less effectively than RHEB WT resulting in increased BRAF/CRAF Heterodimerization

**Fig. 2:**
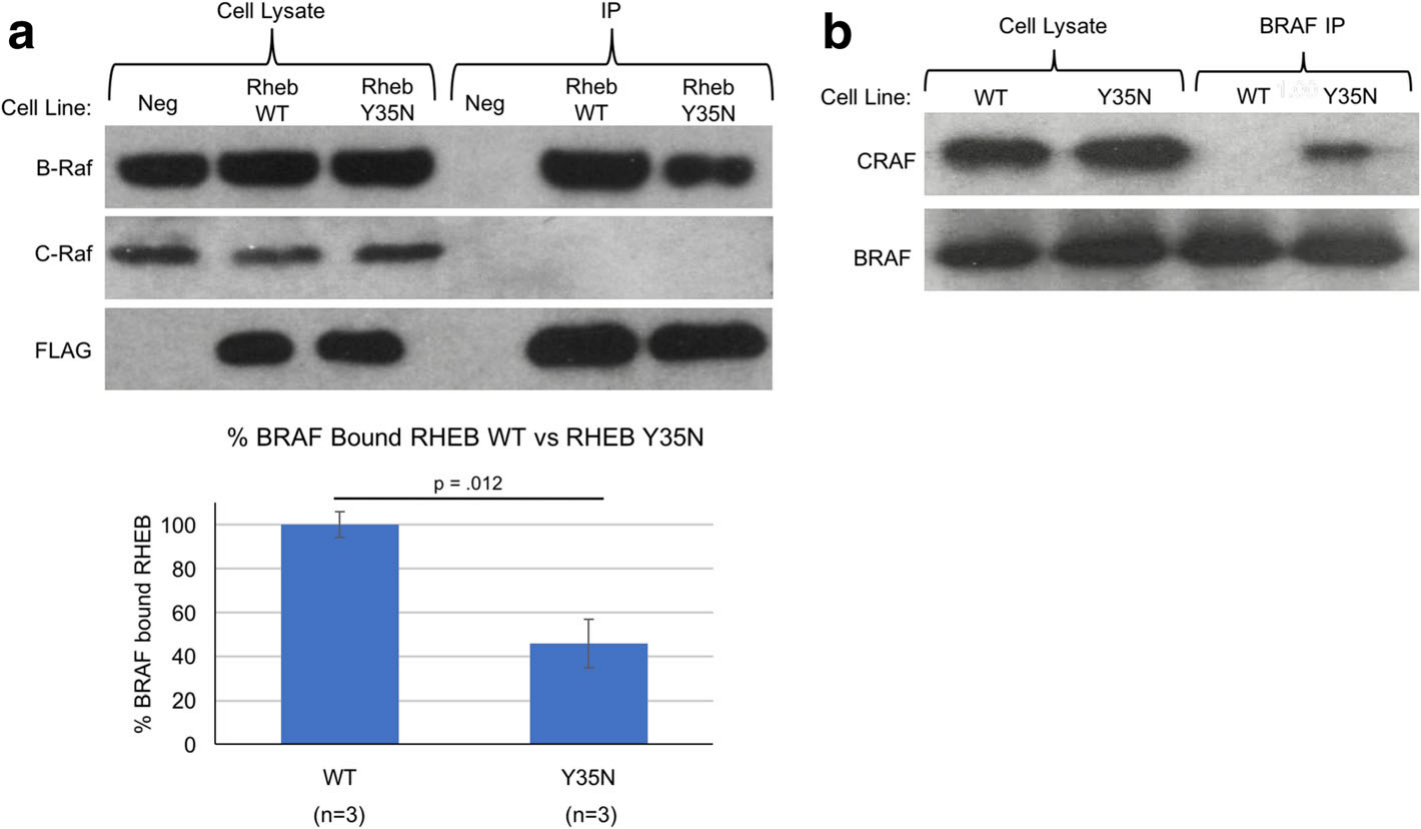
The Y35N Mutation Disrupts RHEB-BRAF Interaction Resulting in Increased BRAF/CRAF Heterodimerization and Activation. (**a**) *Top:* Western blot for BRAF, CRAF, and FLAG is shown. HEK 293T cells were transfected with plasmids expressing FLAG-RHEB WT, FLAG-RHEB Y35N, or an empty plasmid expressing no protein (Neg). Cell Lysate was collected 48 h post transfection, and an immunoprecipitation (IP) using anti-FLAG antibody was carried out. *Bottom:* Graph showing the percentage of BRAF bound RHEB Y35N compared to RHEB WT. A BRAF/RHEB ratio was determined for RHEB WT and for RHEB Y35N using ImageJ to calculate the Western blot band intensities of BRAF and FLAG-RHEB as seen in Western blot above. The BRAF/RHEB ratio for RHEB WT was set to 100%, and RHEB Y35N was normalized to RHEB WT. The graph depicts the results from three separate experiments. **b** Cell lysates were collected from NIH 3T3 cell lines stably expressing RHEB WT or RHEB Y35N. Immunoprecipitation of endogenous BRAF was performed from these lysates. Western blots against CRAF and BRAF are shown. The cell line used for BRAF IP is indicated above the figure as WT (RHEB WT) or Y35N (RHEB Y35N)

An oncogenic mutant of RHEB was identified from the analysis of human cancer genome databases [11]. It has recently been reported that RHEB Y35N transforms cells through and activation of ERK was detected [12]. Since RAF kinase is upstream of RAF/MEK/ERK, we postulated that the Y35N mutation could affect the RHEB-BRAF interaction leading to changes in RAF/MEK/ERK activation. We expressed FLAG-tagged RHEB WT or RHEB-Y35N in HEK293T cells, collected the cell lysates, and performed an immunoprecipitation using anti-FLAG antibody. Western blot analysis using BRAF and CRAF antibodies revealed that RHEB Y35N binds BRAF less effectively than RHEB WT (Fig. 2a).

We observed decreased BRAF-CRAF dimerization in the presence of RHEB WT due to strong RHEB-BRAF interaction, thus it is possible that the decreased RHEB Y35N-BRAF interaction allows for greater BRAF-CRAF dimerization. To test this, we performed immunoprecipitation of BRAF in NIH 3 T3 cell lines stably expressing RHEB Y35N, followed by western blot for CRAF. We see robust BRAF-CRAF heterodimerization in the RHEB Y35N cell lines compared with the RHEB WT cell lines (Fig. 2b). We also observed less RHEB Y35N-BRAF interaction compared with RHEB WT, similar to our results from HEK293T cells (Additional file 1: Figure S2).

### RHEB Y35N activates ERK signaling

**Fig. 3:**
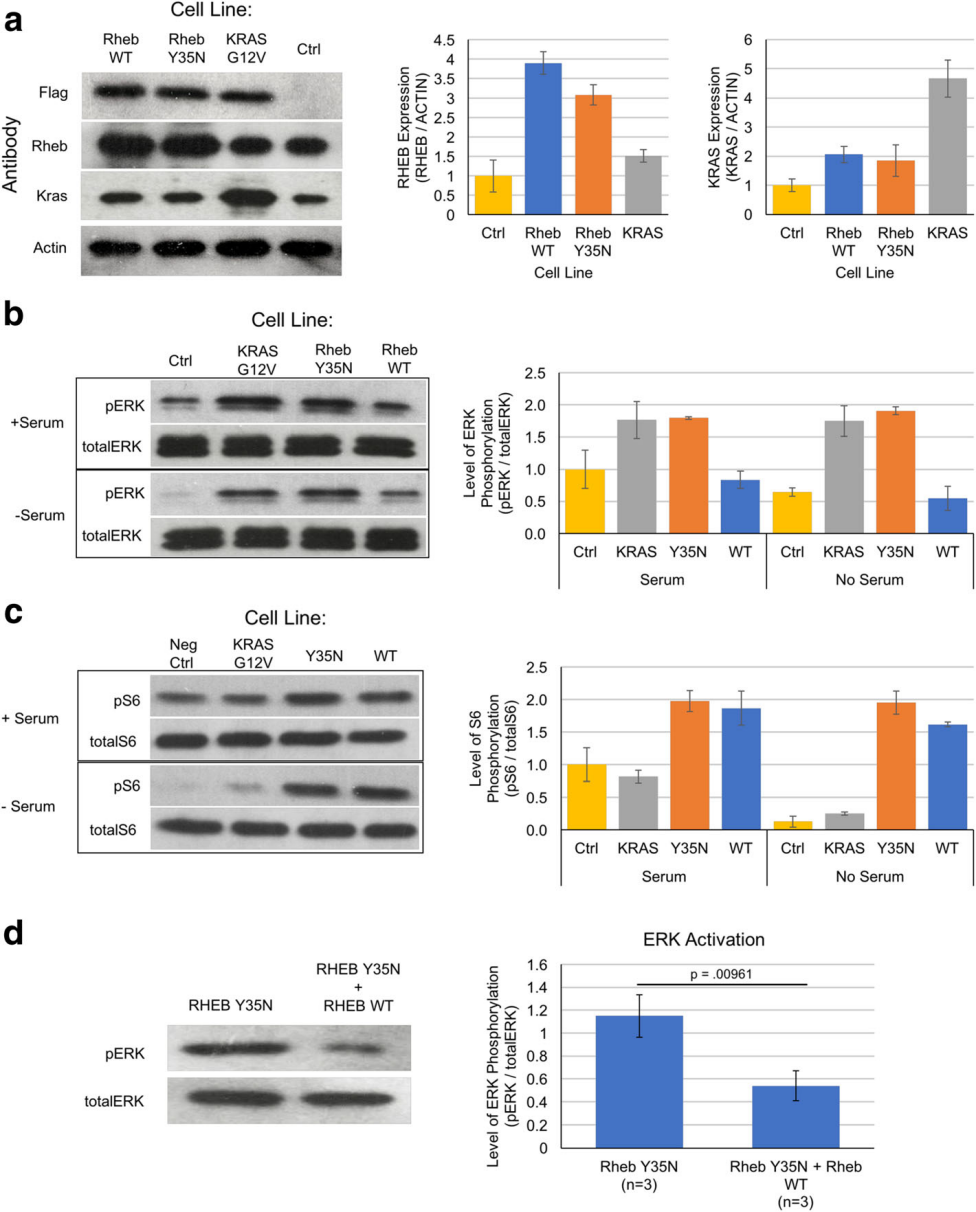
Cell Lines Stably Expressing RHEB Y35N Activate RAF/MEK/ERK Signaling Similar to KRAS G12 V Cell Lines. (**a**) NIH 3 T3 cell lines stably expressing RHEB WT, RHEB Y35N, or KRAS G12V were generated using lentiviral transduction. *Left:* Western blot of FLAG, RHEB, KRAS, and ACTIN protein in total cell lysates collected from NIH3T3 cell lines stably expressing RHEB WT, RHEB Y35N, KRAS G12V, or an empty vector expressing no protein (Ctrl). *Right:* Fold expression for RHEB was calculated by identifying all band intensities using ImageJ analysis, then finding the ratio of RHEB/ACTIN for each sample. The Ctrl sample RHEB/ACTIN ratio was set at 1. All other samples’ ratios were normalized to Ctrl. KRAS fold expression was calculated same as RHEB. Graph represents the averages of experiments (*n* = 2). **b** NIH 3T3 cell lines stably expressing RHEB WT, RHEB Y35N, KRAS G12 V, or Ctrl were grown in the presence or absence of serum, and cell lysates were collected after 24 h. Western blot was performed using antibodies for phosphorylated –ERK, and total –ERK. Fold expression for pERK was calculated as in (**a**). Briefly, band intensities as determined by ImageJ were used to find the ratio of pERK/total ERK for each cell line and condition. The ratio for Ctrl under normal growth conditions was set to 1, and all other ratios were normalized to Ctrl under normal growth conditions. Graph represents the averages of experiments (n = 2). **c** Same experiment as performed in (**b**), except looking at phosphorylated –S6, and total –S6. Fold expression for pS6 was calculated as in (**b**). **d** NIH 3 T3 cell lines stably expressing Y35N were transiently transfected with plasmids to overexpress RHEB WT. *Left:* Western blot for phosphorylated-ERK (pERK) is shown. *Right:* Graph showing ERK activation based on levels of phosphorylated ERK compared to total ERK. The pERK/totalERK ratio for RHEB Y35N and RHEB Y35N + RHEB WT cell lines, was determined by quantifying the intensities of the Western blot bands for pERK and totalERK using imageJ. The graph shows results from three separate experiments

To test whether stable expression of RHEB Y35N activates ERK signaling, we generated NIH 3T3 cell lines stably overexpressing RHEB Y35N. As controls, we also generated cell lines stably overexpressing RHEB WT and KRAS G12V, a strong activator of RAF/MEK/ERK signaling. All three cell lines exhibit a 3–4-fold increase in RHEB or KRAS expression compared to control cell lines (Fig. 3a). We grew the cell lines under normal and serum starved conditions, collected cell lysates, and performed Western blot for phosphorylated and total ERK protein. In the absence of serum, RAF/MEK/ERK should be shut down and we expect to see low levels of phosphorylated ERK. However, the RHEB Y35N cell line showed strong activation of the RAF/MEK/ERK pathway in the absence of serum, similar to the KRAS G12 V cell line (Fig. 3b). The RHEB WT cell line also showed slightly elevated levels of phosphorylated ERK, but at much lower levels than RHEB Y35N or KRAS G12 V cells. This is likely due to increased KRAS expression in the RHEB WT cell lines (Fig. 3a). Additionally, in the same experiment we saw RHEB Y35N cell lines activated mTORC1 signaling similar to RHEB WT in the absence of serum (Fig. 3c).

We hypothesized that the RHEB Y35N mutant activates BRAF in cells through less effective binding, while RHEB WT binds BRAF stronger and inhibits BRAF signaling. We tested whether overexpression of RHEB WT in the RHEB Y35N stably expressing cell line would decrease RAF/MEK/ERK pathway. We transiently transfected RHEB WT into the RHEB Y35N expressing cell lines and monitored changes in levels of phosphorylated-ERK. The expression of RHEB WT in RHEB Y35N cell lines resulted in a significant decrease of phosphorylated-ERK (Fig. 3d). This confirms that RHEB Y35N activates ERK, while RHEB WT shuts it down.

### RHEB Y35N transforms cells

We next looked at the ability of RHEB Y35N to transform normal cells into cancer cells. NIH 3T3 cells were chosen in part because of their sensitivity to Ras mutant transformation and ease of transfection [38]. Transformation of normal cells into cancer cells is characterized by examining the following attributes: reduced serum dependence, loss of density-dependent growth inhibition, and acquisition of anchorage-independent growth [39].

**Fig. 4:**
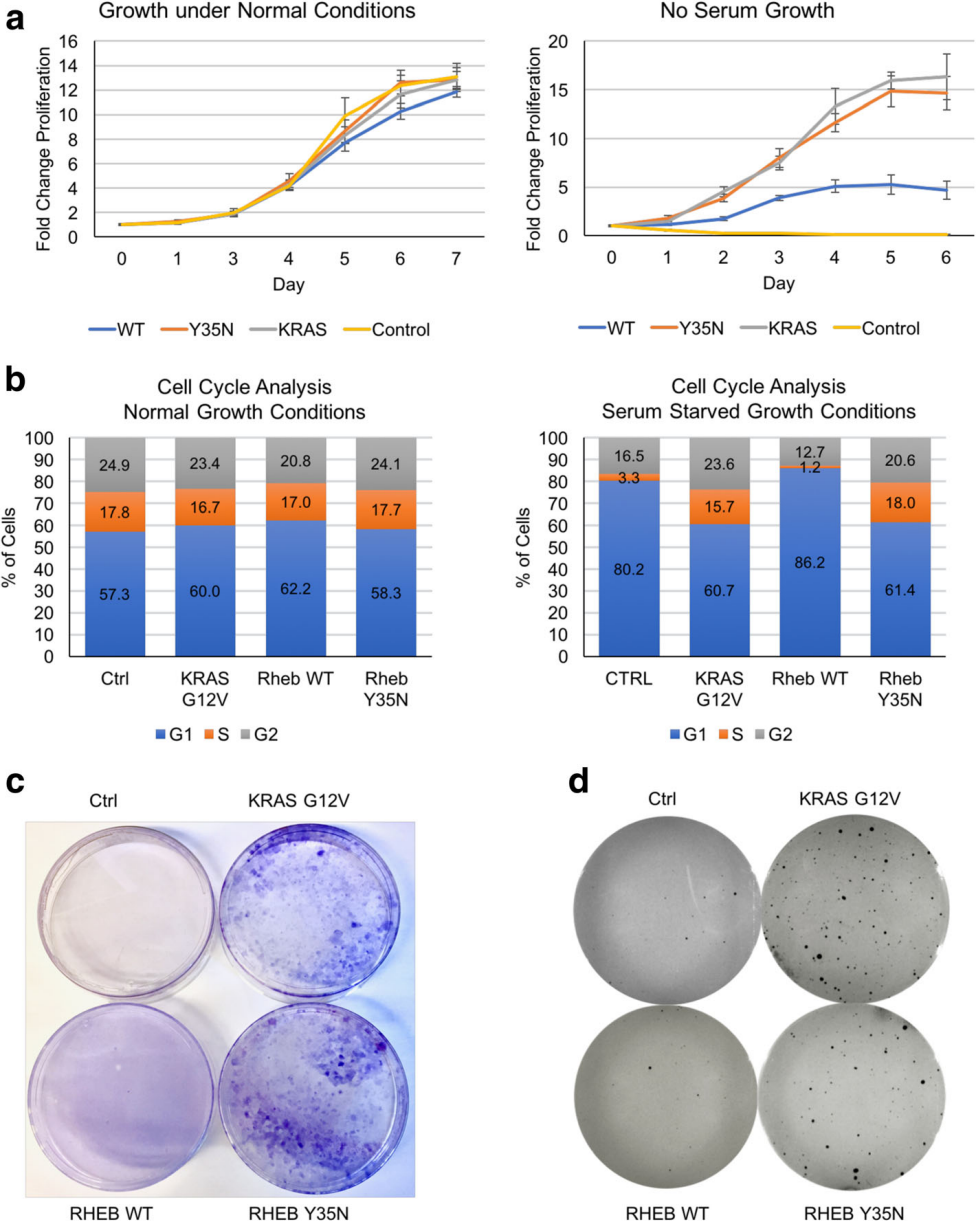
RHEB Y35N Transforms Cells Similar to KRAS G12V and RHEB WT Does Not. (**a**) Growth curves of NIH 3T3 cell lines stably expressing RHEB WT, RHEB Y35N, or KRAS G12 V grown in media containing 10% FBS (left), or serum-free media (right), for 7 days. Fold Growth was calculated as follows: (OD at day X) / (OD at day 0), where OD was read according to Cell Counting Kit-8 (Dojindo Mol. Tech.) protocol. Error bars are the standard deviation measured from three separate experiments. **b** NIH 3T3 stably expressing cell lines were grown for 2 days with serum (left) or without serum (right). Cells were then fixed, treated with RNase A to remove RNA, and incubated with propidium iodide (PI) to dye DNA. Cells were grouped into cell cycle stage based on PI intensity measured using flow cytometry. Graphs show the percentages of cells at each stage in the cell cycle. **c** Foci Formation Assay. NIH 3T3 stably expressing cell lines were grown under low serum conditions for 3 weeks. Cells were fixed with methanol and stained with crystal violet dye for easy visualization. **d** Soft Agar Colony Formation Assay. NIH 3T3 stably expressing cell lines were grown in agar-media suspension for 3 weeks. Plates were incubated overnight with Nitroblue Tetrazolium Chloride (NBT) in order to visualize colonies on a gel imager

Under normal growth conditions, all cell lines showed similar growth curves, however, under serum starved conditions RHEB Y35N cells grew significantly better than RHEB WT cells (Fig. 4a). RHEB Y35N cell lines appear to have a growth curve very similar to the KRAS G12V cell lines, indicative of transformed cancer cell lines.

In addition, RHEB Y35N and KRAS G12V cell lines progress through the cell cycle in the absence of serum, while RHEB WT cell lines do not (Fig. 4b). FACS analysis was carried out to examine cells in different phases of cell cycle. While all cell lines displayed similar percentages of cells in the G1/G2/S phases under normal conditions, only the RHEB WT and Control cell lines arrested in the G1 phase under serum starvation (Fig. 4b, Additional file 2: Figure S3). The KRAS G12V and RHEB Y35N cell lines did not arrest in the G1 phase under serum starvation, and appeared to have similar percentages of cells in the G1/G2/S phases as when grown under normal conditions (Fig. 4b, Additional file 2: Figure S3).

Cellular transformation was evaluated by two different assays. First, we performed a foci formation assay to test the ability of the cell lines to grow in multilayers after several weeks of growth. We observed extensive foci formation in our RHEB Y35N cell lines similar to KRAS G12V (Fig. 3c). Second, we performed a colony formation assay in soft agar to test the ability of the cell lines for anchorage independent growth. We observed a large number of colonies in both the RHEB Y35N and KRAS G12V cell lines (>60), and a low number of colonies in the control and RHEB WT cell lines (<10) (Fig. 4d). These experiments show that RHEB Y35N transforms normal cells into cancer cells similar to KRAS G12V.

### RHEB Y35N transforms cells through RAF/MEK/ERK pathway

**Fig. 5:**
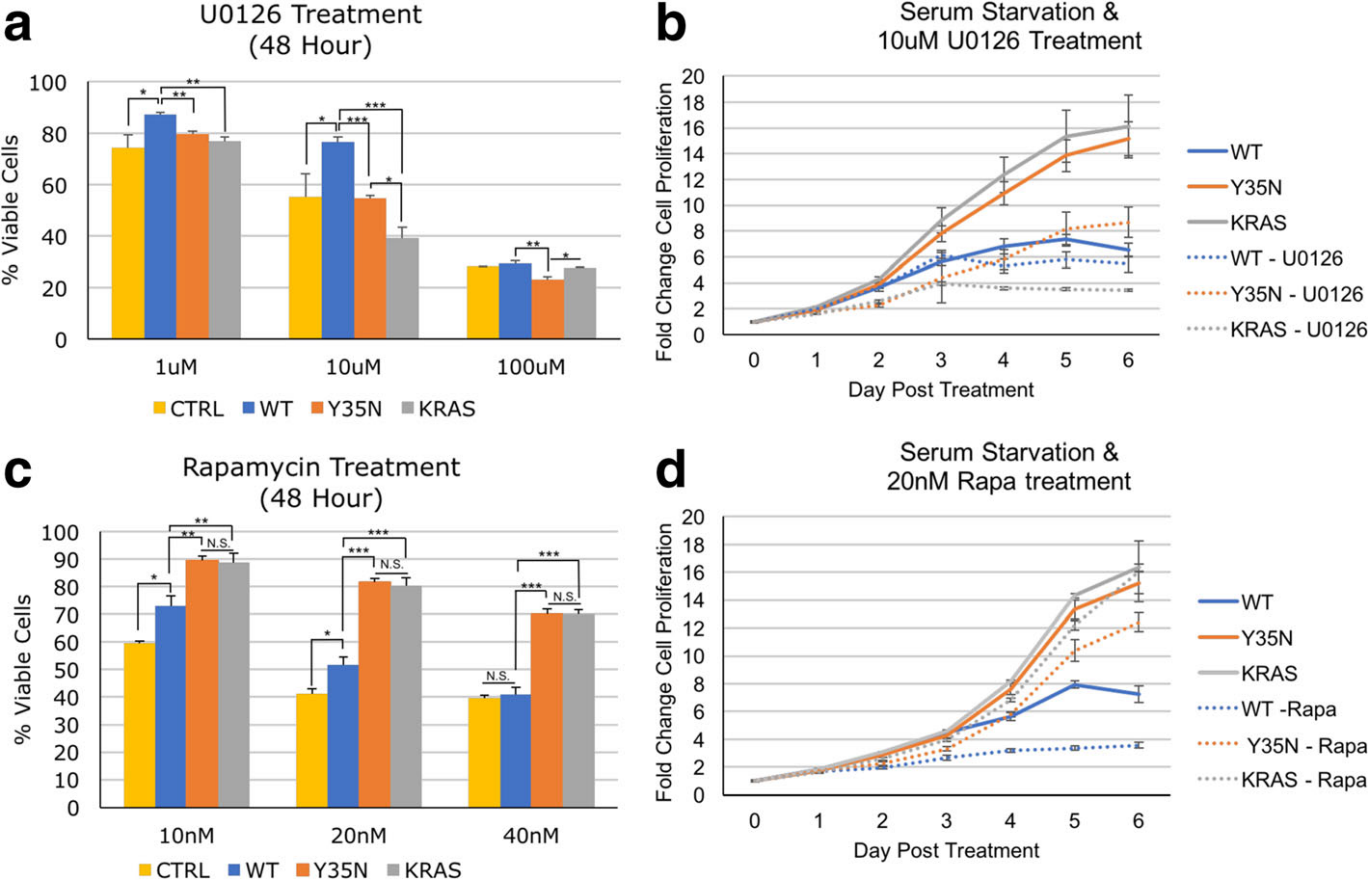
RHEB Y35N is Dependent on RAF/MEK/ERK Signaling, not mTORC1 Signaling, for Proliferation in Low Serum Conditions. (**a**) NIH 3T3 cell lines stably expressing RHEB WT, RHEB Y35N, or KRAS G12V as well as control were treated with 3 different concentrations of RAF/MEK/ERK inhibitor, U0126, for 48 h. % Viable Cells = (OD value of treated cells) / (OD value of non-treated cells) * 100. OD values were measured using Cell Counting Kit-8. Error bars represent standard deviation from three separate experiments, *p < 0.05, **p < 0.01, *** *p* < 0.001. The four cell lines grow similarly under normal condition as shown in Fig. 4a. **b** NIH 3T3 cell lines were grown in serum-free conditions, with or without 10 μM U0126 treatment. Growth was monitored using Cell Counting Kit-8 for 6 days. Error bars are shown from three repeated experiments (**c**) RHEB Y35N growth is not sensitive to mTORC1 inhibition. NIH 3T3 cell lines were treated with 3 different concentrations of mTORC1 inhibitor, Rapamycin, for 48 h. % Viable Cells and statistics calculated as in (**a**). **d** NIH 3T3 cell lines were grown in serum-free conditions, with or without 20 nM Rapamycin treatment. Growth was monitored as in (**b**)

Due to the decreased binding with BRAF, the activation of ERK, and the similar cancer transformation properties to KRAS G12V, we wanted to test whether RHEB Y35N transforms cells through the RAF/MEK/ERK pathway. Using a potent MEK inhibitor (U0126) we see that RHEB Y35N and KRAS G12V cell viability is significantly decreased after 48 h of treatment, whereas RHEB WT cells are less effected (Fig. 5a). Additionally, the growth curves of RHEB Y35N and KRAS G12V are significantly decreased in the presence of 10 μM U0126, while RHEB WT cell lines are much less affected (Fig. 5b). In similar experiments using the mTORC1 inhibitor rapamycin, we observed RHEB Y35N growth was not greatly affected (Fig. 5c and d). This suggests RHEB Y35N transforms cells through the RAF/MEK/ERK pathway independent of mTOR signaling.

## Discussion

In this paper, we have shown that RHEB interacts with BRAF. Use of two RHEB mutants, T38A and D60I established that this interaction is dependent on the intact effector domain as well as on GTP binding status. Thus, BRAF is a downstream effector of RHEB. On the other hand, we have not detected interaction of RHEB with CRAF, suggesting that RHEB specifically interacts with BRAF. The reason RHEB binds BRAF and not CRAF needs further investigation. BRAF and CRAF are very similar in homology, with only a few differences between them. Most notably, BRAF has an extended portion of the N-terminus that is not present in CRAF. It has been reported that this extra N-terminal sequence facilitates RAS binding with BRAF differently than with CRAF [40]. It could be that this is the area where RHEB interacts, but further studies are needed to determine the RHEB binding site on BRAF. We further showed that RHEB inhibits BRAF-CRAF dimer formation.

Significance of RHEB-BRAF interaction was further supported by the experiment to knockdown RHEB. Increased ERK signaling was observed when RHEB expression was inhibited by shRNA. In contrast, overexpression of RHEB results in the inhibition of the ERK signaling. Thus, RHEB suppresses the ERK signaling through its interaction with BRAF and inhibition of the formation of BRAF-CRAF heterodimer.

The oncogenic RHEB mutant Y35N was identified in human cancers including renal cancer and endometrial cancer [11]. We have shown the oncogenic RHEB mutant, RHEB Y35N, interacts less efficiently with BRAF when compared with the wild type RHEB. Furthermore, the Y35N mutant does not inhibit BRAF-CRAF heterodimerization, while the wild type RHEB does. Thus, ERK signaling is sustained at a higher level in mutant cells than in wildtype, contributing to transformation. On the other hand, the RHEB Y35N mutant behaves similarly to the wild type with respect to the activation of mTORC1 signaling. We also examined the binding of the mutant RHEB Y35N to AMPK, as a previous report suggested that RHEB Y35N transforms cancer cells through an interaction with AMPK [12]. This paper argues that RHEB Y35N displays stronger binding to AMPK, which prevents AMPK from phosphorylating and inhibiting BRAF. However, in our experiments we did not observe increased binding of RHEB Y35N to AMPK when compared with the RHEB WT (Additional file 3: Figure S1).

Transforming capability of the RHEB Y35N mutant was evaluated by establishing a stable cell line expressing the mutant RHEB. We find that these cells exhibit serum independent growth; they avoid G1 cell cycle arrest and continue to grow in the absence of serum. These cells also exhibit foci formation and soft agar growth demonstrating anchorage independent growth. Strikingly, the transforming ability of the RHEB mutant was as strong as that of the KRAS G12V mutant. In further support of the significance of the increased ERK signaling and not mTORC1 signaling in the Y35N expressing cells, proliferation of these cells were inhibited by MEK inhibitor but not by rapamycin.

Presence of multiple downstream effectors is a common feature of the Ras superfamily GTPases, as evidenced by identification of multiple downstream effectors of RAS that includes RAF, PI3K, RalGDS, RIN1 and PKC. Our current study firmly establishes that BRAF is a critical downstream effector of RHEB. Since it has been established that mTORC1 is a downstream effector of RHEB, RHEB affects multiple downstream signaling pathways. Further work on RHEB signaling could define the significance of these downstream signaling pathways and in turn define the function of RHEB GTPase.

## Conclusions

In this paper we report a strong interaction between RHEB and BRAF that results in decreased BRAF-CRAF dimerization and decreased BRAF/MEK/ERK signaling. This relationship is greatly affected by the Y35N point mutation, which results in cellular cancer transformation due to decreased RHEB Y35N-BRAF interaction and increased BRAF-CRAF dimerization. This evidence shows a crucial role RHEB has in directly regulating the RAF/MEK/ERK pathway from aberrant activation, provides results that deepen our understanding of RHEB signaling.

## Additional files

Additional file 1: Figure S2. RHEB Y35N Exhibits Decreased Binding to BRAF. Cell lysates were collected from NIH 3T3 cell lines stably expressing FLAG-RHEB WT or FLAG-RHEB Y35N. Immunoprecipitation of endogenous BRAF was performed from these lysates. Western blots against BRAF and FLAG are shown. The cell line used for BRAF IP is indicated above the figure as WT (RHEB WT) or Y35N (RHEB Y35N) (PDF 161 kb)

Additional file 2: Figure S3. Flow Cytometry Data for Cell Cycle Analysis. NIH 3T3 cell lines stably expressing FLAG-RHEB WT or FLAG-RHEB Y35N were grown for 2 days with serum (normal growth, top row) or without serum (serum starved, bottom row). Cells were then fixed, treated with RNase A to remove RNA, and incubated with propidium iodide (PI) to dye DNA. Cells were grouped into cell cycle stage based on PI intensity measured using flow cytometry. Flow cytometry statistics for each sample is shown to the right of each graph (PDF 434 kb)

Additional file 3: Figure S1. RHEB Y35N Does not Exhibit Increased Binding to AMPK. A) RHEB WT, T38A, and Y35N mutants were transiently transfected and expressed in HEK 293T cells, cell lysates were collected, and immunoprecipitation for each was carried out. These results show a Western blot for AMPKα and FLAG from those samples. An effector domain mutant, RHEB T38A, did not bind AMPK demonstrating that AMPK is a relevant effector of RHEB (PDF 154 kb)

## Abbreviations

AMPK: AMP-Activated Protein Kinase
ERK: Extracellular Signal-Related Kinase
MEK: Mitogen-Activated Protein Kinase Kinase
mTORC1: Mechanistic Target of Rapamycin Complex 1
RAF: Rapidly Accelerated Fibrosarcoma
RHEB: Ras Homolog Enriched in Brain
shRNA: Short Hairpin RNA
