# Supplementary figures and images for: An oncogenic mutant of RHEB, RHEB Y35N, exhibits an altered interaction with BRAF resulting in cancer transformation
Source: BMC Cancer. 2018 Jan 10;18:69. doi: 10.1186/s12885-017-3938-5 (PMC5763582; doi:10.1186/s12885-017-3938-5)

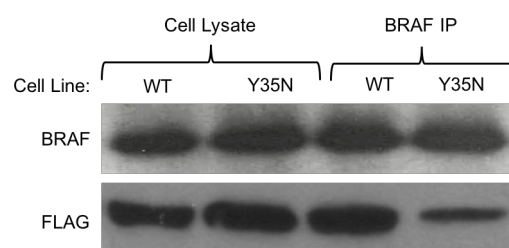

Supplement: Supplementary file 1 — RHEB Y35N Exhibits Decreased Binding to BRAF. Cell lysates were collected from NIH 3T3 cell lines stably expressing FLAG-RHEB WT or FLAG-RHEB Y35N. Immunoprecipitation of endogenous BRAF was performed from these lysates. Western blots against BRAF and FLAG are shown. The cell line used for BRAF IP is indicated above the figure as WT (RHEB WT) or Y35N (RHEB Y35N) (PDF 161 kb) [file 12885_2017_3938_MOESM1_ESM.pdf]

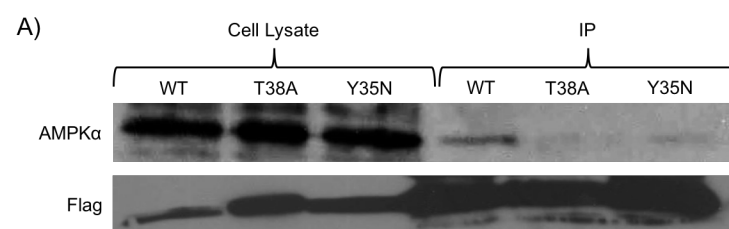

Supplement: Supplementary file 3 — RHEB Y35N Does not Exhibit Increased Binding to AMPK. A) RHEB WT, T38A, and Y35N mutants were transiently transfected and expressed in HEK 293T cells, cell lysates were collected, and immunoprecipitation for each was carried out. These results show a Western blot for AMPKα and FLAG from those samples. An effector domain mutant, RHEB T38A, did not bind AMPK demonstrating that AMPK is a relevant effector of RHEB (PDF 154 kb) [file 12885_2017_3938_MOESM3_ESM.pdf]
