# Supplementary material for: An oncogenic mutant of RHEB, RHEB Y35N, exhibits an altered interaction with BRAF resulting in cancer transformation
Source: BMC Cancer. 2018 Jan 10;18:69. doi: 10.1186/s12885-017-3938-5 (PMC5763582; doi:10.1186/s12885-017-3938-5)
Supplement: Supplementary file 2 — Flow Cytometry Data for Cell Cycle Analysis. NIH 3T3 cell lines stably expressing FLAG-RHEB WT or FLAG-RHEB Y35N were grown for 2 days with serum (normal growth, top row) or without serum (serum starved, bottom row). Cells were then fixed, treated with RNase A to remove RNA, and incubated with propidium iodide (PI) to dye DNA. Cells were grouped into cell cycle stage based on PI intensity measured using flow cytometry. Flow cytometry statistics for each sample is shown to the right of each graph (PDF 434 kb) [file 12885_2017_3938_MOESM2_ESM.pdf]

Rheb WT Normal Growth

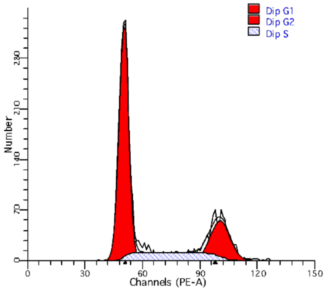

File analyzed: Specimen_001_Tube_
Date analyzed: 31-Jan-2017
Model: 1nn0n_DSF
Analysis type: Manual analysis

Ploidy Mode: First cycle is diploid

Diploid: 100.00 %
Dip G1: 62.22 % at 50.37
Dip G2: 20.83 % at 100.58
Dip S: 16.95 % G2/G1: 2.00
%CV: 5.02

Total S-Phase: 16.95 %
Total B.A.D.: 0.00 % no debris no a

Debris: %
Aggregates: 0.00 %
Modeled events: 3355
All cycle events: 3355
Cycle events per channel: 66
RCS: 0.937

Rheb Y35N Normal Growth

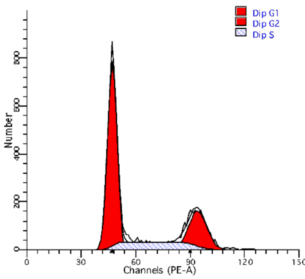

File analyzed: Specimen_001_Tube_
Date analyzed: 31-Jan-2017
Model: 1nn0n_DSF
Analysis type: Manual analysis

Ploidy Mode: First cycle is diploid

Diploid: 100.00 %
Dip G1: 58.29 % at 47.18
Dip G2: 24.05 % at 94.05
Dip S: 17.66 % G2/G1: 1.99
%CV: 5.33

Total S-Phase: 17.66 %
Total B.A.D.: 0.00 % no debris no a

Debris: %
Aggregates: 0.00 %
Modeled events: 8393
All cycle events: 8393
Cycle events per channel: 175
RCS: 1.793

Kras G12V Normal Growth

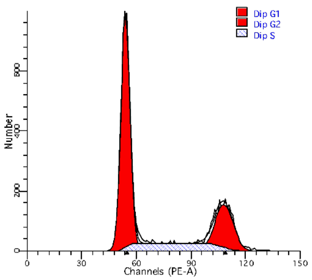

File analyzed: Specimen_001_Tube_
Date analyzed: 31-Jan-2017
Model: 1nn0n_DSF
Analysis type: Manual analysis

Ploidy Mode: First cycle is diploid

Diploid: 100.00 %
Dip G1: 59.97 % at 54.13
Dip G2: 23.36 % at 108.15
Dip S: 16.67 % G2/G1: 2.00
%CV: 4.64

Total S-Phase: 16.67 %
Total B.A.D.: 0.00 % no debris no a

Debris: %
Aggregates: 0.00 %
Modeled events: 8325
All cycle events: 8325
Cycle events per channel: 151
RCS: 1.767

Ctrl Normal Growth

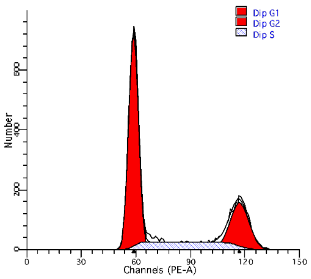

File analyzed: Specimen_001_Tube_
Date analyzed: 31-Jan-2017
Model: 1nn0n_DSF
Analysis type: Manual analysis

Ploidy Mode: First cycle is diploid

Diploid: 100.00 %
Dip G1: 57.28 % at 58.81
Dip G2: 24.92 % at 116.99
Dip S: 17.80 % G2/G1: 1.99
%CV: 4.34

Total S-Phase: 17.80 %
Total B.A.D.: 0.00 % no debris no a

Debris: %
Aggregates: 0.00 %
Modeled events: 8119
All cycle events: 8119
Cycle events per channel: 137
RCS: 1.049

Rheb WT Serum Starved

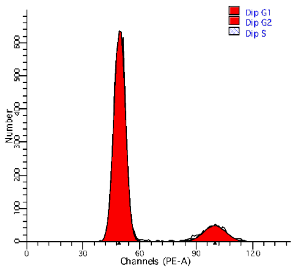

File analyzed: Specimen_001_Tube_
Date analyzed: 31-Jan-2017
Model: 1nn0n_DSF
Analysis type: Manual analysis

Ploidy Mode: First cycle is diploid

Diploid: 100.00 %
Dip G1: 86.16 % at 49.47
Dip G2: 12.68 % at 99.88
Dip S: 1.16 % G2/G1: 2.02
%CV: 5.84

Total S-Phase: 1.16 %
Total B.A.D.: 0.00 % no debris no a

Debris: %
Aggregates: 0.00 %
Modeled events: 5434
All cycle events: 5434
Cycle events per channel: 106
RCS: 0.957

Rheb Y35N Serum Starved

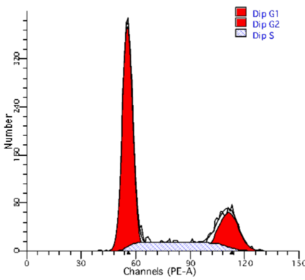

File analyzed: Specimen_001_Tube_
Date analyzed: 31-Jan-2017
Model: 1nn0n_DSF
Analysis type: Manual analysis

Ploidy Mode: First cycle is diploid

Diploid: 100.00 %
Dip G1: 61.35 % at 55.60
Dip G2: 20.63 % at 111.15
Dip S: 18.02 % G2/G1: 2.00
%CV: 4.99

Total S-Phase: 18.02 %
Total B.A.D.: 0.00 % no debris no a

Debris: %
Aggregates: 0.00 %
Modeled events: 4306
All cycle events: 4306
Cycle events per channel: 76
RCS: 0.675

Kras G12V Serum Starved

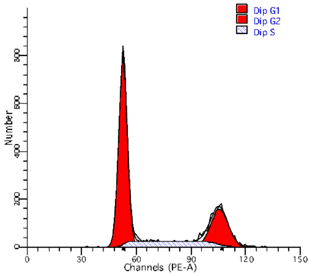

File analyzed: Specimen_001_Tube_
Date analyzed: 31-Jan-2017
Model: 1nn0n_DSF
Analysis type: Manual analysis

Ploidy Mode: First cycle is diploid

Diploid: 100.00 %
Dip G1: 60.65 % at 52.76
Dip G2: 23.64 % at 105.70
Dip S: 15.71 % G2/G1: 2.00
%CV: 4.50

Total S-Phase: 15.71 %
Total B.A.D.: 0.00 % no debris no a

Debris: %
Aggregates: 0.00 %
Modeled events: 7979
All cycle events: 7979
Cycle events per channel: 148
RCS: 1.135

Ctrl Serum Starved

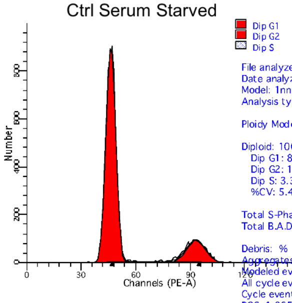

File analyzed: Specimen_001_Tube_008_008.fcs
Date analyzed: 31-Jan-2017
Model: 1nn0n_DSF
Analysis type: Manual analysis

Ploidy Mode: First cycle is diploid

Diploid: 100.00 %
Dip G1: 80.20 % at 46.38
Dip G2: 16.46 % at 93.28
Dip S: 3.34 % G2/G1: 2.01
%CV: 5.48

Total S-Phase: 3.34 %
Total B.A.D.: 0.00 % no debris no aggs

Debris: %
Aggregates: 0.00 %
Modeled events: 7195
All cycle events: 7195
Cycle events per channel: 150
RCS: 1.065
